# Emotion-focused therapy as a transdiagnostic treatment for depression, anxiety and related disorders: Protocol for an initial feasibility randomised control trial

**DOI:** 10.12688/hrbopenres.12993.1

**Published:** 2020-02-13

**Authors:** Ladislav Timulak, Daragh Keogh, James McElvaney, Sonja Schmitt, Natalie Hession, Katarina Timulakova, Ciaran Jennings, Fiona Ward

**Affiliations:** 1 School of Psychology, Trinity College Dublin, Dublin, 2, Ireland; 2 Institute of Emotion-Focused Therapy Ireland, Dublin, 2, Ireland; 3 St. Luke's Hospital, Dublin, Dublin, Ireland; 4 St. Vincent's Hospital, Fairview, Dublin, Ireland; 5 South Dublin Counselling & Psychological Services, Dublin, Ireland; 6 HSE National Counselling Service, Dublin, Ireland

**Keywords:** Emotion-focused therapy, transdiagnostic, EFT-T, depression, anxiety, protocol

## Abstract

**Background: **Depression, anxiety and related disorders, including obsessive-compulsive disorders and trauma/stressor related disorders, have high prevalence, chronic courses and cause significant impairment. These disorders are also highly co-morbid, and appear to share etiology and maintenance factors. Recent developments have seen the emergence of transdiagnostic approaches that systematically address the common/shared features of these disorders. A key advantage of transdiagnostic approaches is that they can reduce the pressure on mental health professionals to be proficient in a plethora of single-disorder focused treatments. Currently almost all transdiagnostic approaches come from cognitive-behavioural therapy (CBT). However, not all clients prefer or benefit from CBT. Emotion-focused therapy (EFT) represents an evidence-based alternative to CBT. This study aims to examine a transdiagnostic adaptation of EFT (EFT-T) as a treatment for depression, anxiety and related disorders.

**Method:** The current study is a randomised controlled trial that aims to establish the efficacy of EFT-T vs. wait-list control in the treatment of depression, anxiety and related disorders. Up to 40 clients presenting in a psychology/counselling service will be randomly assigned to two conditions: EFT-T (n=20) and wait-list control, with delayed intervention (n=20). Primary outcome measures will be the Overall Anxiety Severity and Impairment Symptoms, the Overall Depression Severity and Impairment Symptoms, and the Clinical Outcome in Routine Evaluation – Outcome Measure. Disorder specific self-report measures will also be used to assess the main symptomatology of respective primary diagnoses. Clients will be assessed prior to therapy, at week 16, at end of therapy, and at 6 months follow-up.

**Discussion:** This study aims to provide an initial test of EFT-T as a transdiagnostic treatment for depression, anxiety and related disorders. It will provide estimates of effects sizes that can inform power calculations for a comparative trial, comparing EFT-T to a standard transdiagnostic treatment, CBT.

**Registration:** ISRCTN11430110; registered on 07 January 2019.

## Abbreviations

APA: American Psychiatric Association; CBT: Cognitive-behavioural therapy; CCIP: Client Change Interview Protocol; CORE-OM: Clinical Outcome in Routine Evaluation – Outcome Measure; DSM: Diagnostic and Statistical Manual of Mental Disorders; EFT: Emotion-focused therapy; EFT-T: Transdiagnostic emotion-focused therapy; GAD: Generalised anxiety disorder; GAD-7: Generalized Anxiety Disorder-7; GP: General Practitioner; HSE: Health Service Executive; ITT: Intent-to-treat; LSAS: Liebowitz Social Anxiety Scale; MDD: Major depressive disorder; MEDI: Multidimensional Emotional Disorder Inventory; OASIS: Overall Anxiety Severity and Impairment Scale; ODSIS: Overall Depression Severity and Impairment Scale; OFSIS: Overall Fear Severity and Impairment Scale; OLSIS: Overall Loneliness Severity and Impairment Scale; OSSIS: Overall Shame Severity and Impairment Scale; PCEPS-EFT: Person-Centered and Experiential Psychotherapy Scale - Emotion-Focused Therapy version; PCL-5: PTSD Checklist for DSM-5; PDD: Persistent depressive disorder; PDSS: Panic Disorder Severity Scale; PHQ-9: Patient Health Questionnaire-9; PI: Principal investigator; PP: Per-protocol; PTSD: Posttraumatic stress disorder; RCT: Randomised controlled trial; SAD: Social anxiety disorder; SCID-5-PD: Structured Clinical Interview for DSM-5 Personality Disorders; SCID-5-RV: Structured Clinical Interview for DSM-5, Research Version; SMA-A: Severity Measure for Agoraphobia - Adult; SMSP-A: Severity Measure for Specific Phobia - Adult; TCD: Trinity College Dublin; TMG: Trial Management Group; TSC: Trial Steering Committee; UP: Unified Protocol; Y-BOCS: Yale-Brown Obsessive-Compulsive Scale.

## Introduction

Over recent decades, the dominant paradigm in psychotherapeutic treatment has been disorder-specific treatment. In particular, a wide-range of evidence-based disorder-specific treatments have been developed to target depression, anxiety, and related disorders such as obsessive-compulsive disorder (OCD) and trauma/stressor related disorders ( American Psychiatric Association [APA], 2013). The focus on developing interventions for these disorders is understandable. Together they account for the majority of presentations typically seen in outpatient or primary care settings. Given the prevalence and impact of these disorders, treatments specifically targeting these disorders would seem to make sense. However, the very proliferation of disorder specific treatments (and their attendant manuals) has been identified as in and of itself a potential barrier to the effective dissemination of evidence-based psychological interventions, requiring clinicians to discern between and become competent at delivering multiple interventions ( Wilamowska *et al.*, 2010). Furthermore, while the aforementioned disorders constitute distinct diagnostic categories, the clinical reality is that comorbid presentations are the norm rather than the exception. For example, Brown *et al.* (2001) reported the current and lifetime prevalence of additional Axis I disorders in principal anxiety and mood disorders as 57% and 81%, respectively. While comorbid presentations can be a diagnostic challenge for clinicians in a single-disorder treatment context (e.g., which disorder should be targeted for treatment), the picture is further complicated by observations that the successful treatment of one disorder can have a positive impact on comorbid disorders ( Kennedy & Barlow, 2018). Furthermore, the growing evidence suggests that distinct disorders may have many factors in common including shared developmental risks ( Côté *et al.*, 2009) and genetic vulnerability (e.g., Middeldorp *et al.*, 2005).

The factors summarised above have led to hypotheses that common psychological processes, such as experiential avoidance, perfectionism, or negative reactions to emotional experience, may underpin various emotional disorders ( Egan *et al.*, 2011; Mansell *et al.*, 2008). On this basis, various research teams have developed transdiagnostic treatments which aim to target these common processes (e.g., Barlow *et al.*, 2017b; Riley *et al.*, 2007). Results from studies examining the effectiveness of such treatments are promising, both in their own right, and when compared to disorder-specific treatments (e.g., Barlow *et al.*, 2017a; Newby *et al.*, 2015).

To date, transdiagnostic interventions has emerged predominantly from within the cognitive behavioural therapy (CBT) paradigm. However, CBT is not the preferred treatment for all clients, nor do all clients benefit from CBT (cf. King *et al.*, 2000), and the development of effective evidence-based transdiagnostic treatments based on paradigms other than CBT has the potential to increase treatment choice for both patient and clinician. Emotion-focused therapy (EFT; Greenberg *et al.*, 1993; Greenberg, 2016) was developed within the tradition of humanistic-experiential therapies as an alternative to CBT. While EFT was developed as a generic approach, it has been extensively studied as a disorder-specific intervention, for example in the contexts of major depressive disorder (MDD; cf. Goldman *et al.*, 2006; Greenberg & Watson, 1998; Watson *et al.*, 2003), complex trauma (e.g., Paivio & Nieuwenhuis, 2001), social anxiety disorder (SAD; Shahar *et al.*, 2017) and generalized anxiety disorder (GAD; Timulak *et al.*, 2017).

The systematic application of EFT to specific mood and anxiety disorders, its adaptability to different diagnostic and client presentations, and our own clinical and research experience with co-morbid presentations (e.g., Timulak *et al.*, 2017; Timulak *et al.*, 2018), led the first two authors of the current paper to become interested in re-conceptualizing and systematizing the various EFT clinical protocols and experiences into a single transdiagnostic approach (hereafter referred to as EFT-T) ( Timulak & Keogh, 2020), and to testing this model (described below) in a feasibility randomized controlled trial. The chosen design (wait-list control with subsequent delayed intervention) is the same as was used in early tests of the unified protocol (UP), the currently best-established CBT transdiagnostic intervention ( Barlow *et al.*, 2017b) thus potentially allowing for preliminary tentative benchmark comparisons between EFT-T and UP.

## Objectives

The current feasibility project will provide first comparison data against a wait-list that, if proven to be promising, should help to plan a trial that would establish the relative efficacy of EFT-T in comparison to CBT as an established transdiagnostic treatment. The current project will test recruitment, adherence, and retention rates, as well as providing estimates of comparative outcomes that can be used to inform power calculations for any comparative trial. It is also envisaged that data will be used for process, process-outcome, case study and qualitative analyses that should further inform the transdiagnostic formulation of EFT.

## Method

### Trial design and setting

The design of the study is a randomised controlled trial (RCT) with participants randomly allocated to one of two groups (EFT-T or wait-list with delayed EFT-T intervention). Participants will be seen in a private counselling clinic in Dublin, Ireland, offering psychological therapy to people with depression, anxiety and related disorders. The trial will be promoted by an advertisement on the hosting clinic’s website and participation will be via general practitioner (GP) referral to the trial.

### Participants/clients

Participants (n=40) will be adults (≥18 years), who have been referred by their GP to the EFT-T project. Clients will be screened for depression and anxiety disorders using the Overall Anxiety Severity and Impairment Scale (OASIS; Norman *et al.*, 2006) and the Overall Depression Severity and Impairment Scale (ODSIS; Bentley *et al.*, 2014). If the client’s score on OASIS and/or ODSIS is ≥8, and a diagnosis of depression (specifically MDD or persistent depressive disorder [PDD]), an anxiety disorder, and/or a related obsessive-compulsive or trauma/stressor related disorder is suspected, a comprehensive assessment will follow using the Structured Clinical Interview for DSM-5 Research Version (SCID-5-RV; First *et al.*, 2015).

Clients who meet the criteria for depression, an anxiety disorder and/or a related obsessive-compulsive or trauma/stressor related disorder as a principal diagnosis, will be assigned to one of the trial arms. Therapy will be provided free of charge. To participate in the study, participants must consent to the conditions of the study, including the audio/video recording of sessions (these will be later used for secondary process, process-outcome, case study and qualitative research) and attendance at pretherapy, post-therapy and 6-month follow-up assessment sessions. Individuals taking psychotropic medication must be stabilised on that medication for 6 weeks prior to commencing participation in the trial (cf. Newman & Llera, 2011). Clients on psychotropic medication will also have to show, with their physician’s approval, a willingness to maintain this stability in medication use during the period of therapy, or where participants are in the waitlist/delayed intervention condition, from the time of assessment though completion of therapy. Medication use will be monitored during the trial.

Participants must also give consent for their GP to be contacted in relation to their participation in the study and/or in relation to concerns that may arise about participant well-being. Exclusion criteria are concurrent psychological one-to-one or group treatment (concurrent participation in a mental health support group is permitted), suicide risk, risk of harm to others, substance abuse, bipolar disorders, psychosis, and organic brain syndrome. All of the above will be determined during the SCID-5-RV assessment. Suicide risk and risk of harm to others will also be determined by scores other than 0 on Item 16 (“I have made plans to end my life”), and Item 6 (“I have been physically violent to others”) on the Clinical Outcome in Routine Evaluation – Outcome Measure (CORE-OM; Evans *et al.*, 2000) (see below). Participants not meeting eligibility criteria will be re-referred back to their GP. For further information on intake assessment see below.

### Therapists

It is envisaged that interventions will be delivered by at least 5 therapists. Therapists will be certified in EFT (meeting standards of the International Society for Emotion-Focused Therapy) and will receive additional training in EFT-T (facilitated by Ladislav Timulak, an EFT trainer accredited by the International Society for Emotion-Focused Therapy). In addition, therapists will attend monthly supervision. Supervision will be provided by Ladislav Timulak.

## Intervention

### Emotion-focused therapy – transdiagnostic (EFT-T)

The EFT-T intervention will follow a recently developed model ( Timulak, 2015; Timulak & Keogh, 2020) integrating EFT adaptations for various disorders (e.g., Elliott & Shahar, 2017; Greenberg & Watson, 2006; Paivio & Pascual-Leone, 2010; Timulak & McElvaney, 2017; Watson & Greenberg, 2017) using a unique transdiagnostic framework. The transdiagnostic model, conceptually based on a model of emotional transformation processes in psychotherapy (see Timulak, 2015 and Timulak & McElvaney, 2017), uses (1) elements of a modular approach targeting symptom level presentations (i.e., with some interventions used in the context of certain primary diagnoses/presentations) and (2) an underlying emotional vulnerability approach that targets the chronic painful and feared emotions theorised as underpinning client presentations in cases of depression, anxiety and related disorders. The EFT-T model uses a specific case conceptualisation which postulates that depression and anxiety symptoms signal difficulty (e.g., dysregulation/avoidance) in processing specific chronic painful feelings (specifically sadness/loneliness, shame, and primary fear/terror) triggered by interactions with the environment. It is these triggers that the client either avoids, through a variety of emotional and/or behavioural avoidance mechanisms, or is distressed about, with this distress ultimately manifesting in the form of depression and anxiety symptoms. It is also postulated that in the context of these triggers, the client often attempts to cope with painful feelings through problematic self-treatment.

The model of transformation proposes that the client is first facilitated to use internal resources to cope with symptoms, and to recognize his or her own agency in contributing to the development of these symptoms. The client is then helped to develop a capacity to access and tolerate the specific painful feelings underlying these symptoms, and by doing so, to identify and articulate the unmet needs embedded in these painful feelings. This in turn allows for a process of emotional transformation whereby the client is ultimately helped to transform maladaptive emotions through the generation of adaptive emotional responses (e.g., compassion or protective anger) to identified unmet emotional needs. Therapy thus focuses on (1) a firm case conceptualization; (2) the provision of an emotionally attuned and compassionate therapeutic relationship; (3) the overcoming of symptoms (e.g., ruminations, worries, obsessions, flashbacks, overwhelming distress) through increasing internal coping resources and through experiential tasks which highlight both the function and cost of the emotional processes which bring about and maintain symptoms; and (4) the transformation of chronic underlying painful maladaptive emotions through experiential tasks that activate maladaptive emotions (loneliness/sadness, shame, fear), bring to awareness the unmet needs (for connection, validation and protection) embedded in those painful emotions, and facilitate the generation of adaptive emotional responses (e.g., compassion and protective anger) to those unmet needs.

Therapy will last 16 to 20 sessions. Therapists will be instructed to finish therapy at session 16, but will have flexibility based on their clinical judgement to extend therapy to a maximum of 20 sessions. This flexible ending is based on learning from a previous project [ Timulak *et al.*, 2017], in which it became apparent that some clients needed more than the initially anticipated number of sessions, but benefited significantly from the addition of a relatively small number of sessions. We propose that therapists extend therapy for up to an additional four sessions if (1) the client continues to be clinically distressed (e.g., the therapist can use a formal assessment such as the OASIS and ODSIS, which is collected as part of the study) and (2) the client expresses an explicit wish to continue with therapy for this duration.

The delayed intervention, offered to wait-list participants at 16 weeks, will be delivered as per the active intervention described above.

### Treatment fidelity assessment

Treatment fidelity, in terms of both adherence to protocol and competence of delivery, will be enhanced through therapist attendance at monthly group supervision, and will be evaluated by means of an independent assessment of a sample of video/audio recordings of sessions using the Person-Centered and Experiential Psychotherapy Scale (EFT version; PCEPS-EFT; Elliott, 2016; Freire *et al.*, 2014). All sessions will be audio/video recorded. It is anticipated that one session will be randomly selected for each case and rated by an independent EFT expert (i.e., a certified EFT therapist and/or supervisor). The first and last two sessions from each case will be excluded from the pool of potential sessions used for rating, as typically these sessions contain less experiential work, and thus are less useful for assessing adherence to EFT. To establish reliability, a portion of sessions will also be rated by at least two independent expert raters.

### Randomisation

A random sequence of numbers 1 and 2, corresponding to EFT-T or wait-list, will be generated using an on-line random number generator for each participating therapist, and these lists will be held by a colleague who is independent of, and blind to, the assessment (and allocation to therapist) process. Post SCID-5-RV assessment (see below), the trial manager will allocate participants to the next available therapist, and then request the assignment (either EFT-T or wait-list) for that participant. All assessments will be carried out by a professional other than the therapist. Steps will be taken to ensure week 16, end of therapy, and 6-month follow-up assessments are carried out by professionals blinded to the condition the client is in.

### Client consent process and assessment

The trial will be advertised on the hosting clinic website. Potential participants interested in taking part in the study will be provided with an email address to contact the trial. On making contact they will be provided with two information sheets about the study, one for themselves as a potential participant, and one for their GP (see Extended data; Timulak, 2020). These information sheets contain relevant information about the study background; eligibility and exclusion criteria; steps required to become involved in the study; assessment process; study/therapy process; potential benefits and risks of participation; confidentiality; consent; right to withdraw from the study; and data management. They provide details about, and contact information for, the research team. The GP Information Sheet also contains a checklist summarising the main points relevant for GPs when considering the appropriateness of referring a patient to the trial (e.g., that in the GP’s judgement, their patient potentially meets criteria for depression, anxiety and/or a related disorder as a principal diagnosis; that it is appropriate for their patient to remain on their current psychotropic medication regime; that it is appropriate for their patient to be allocated to the waitlist condition; that GP is willing to be contact by the research team if there are concerns about participant well-being). Potential participants in receipt of a GP referral to the trial will be invited to contact the research team by email, and the research team will contact the client to schedule the first of two assessment appointments.

During the first assessment appointment, a member of the research team will meet with the potential participant to discuss the study, address any queries arising from perusal of the Information Sheet, and seek consent to proceed with the assessment process. They will then screen the potential participant for the trial by reviewing the GP referral letter and by administering the OASIS and ODSIS self-report measures. Where a potential participant presents with depression, anxiety and/or a related disorder and has a score ≥8 on the OASIS and/or ODSIS, they will be invited to proceed with the assessment process and will be asked to complete the CORE-OM and Multidimensional Emotional Disorder Inventory (MEDI; Rosellini, 2013; Rosellini *et al.*, 2015), an experimental measure used in transdiagnostic research measures. They will then be invited to attend a second assessment session.

The second interview-style assessment will involve administration of the SCID-5-RV and the Structured Clinical Interview for DSM-5 Personality Disorders (SCID-5-PD; First *et al.*, 2016). Where assessment indicates depression, anxiety and/or a related disorder as a principal diagnosis, and where inclusion criteria are confirmed as being met, the client will be invited to participate in the study. Time will then be taken to address any queries the client may have, and the client will be asked to sign the study consent form (see *Extended data*; Timulak, 2020).

Each participant will also be asked to complete a diagnosis specific self-report measure. In the case of a primary diagnosis of depression (MDD or PDD), participants will complete the Patient Health Questionnaire-9 (PHQ-9; Kroenke *et al.*, 2001); in the case of the primary diagnosis being Panic Disorder, participants will be asked to complete the Panic Disorder Severity Scale (PDSS; Shear *et al.*, 1997); in the case of Agoraphobia, the Severity Measure for Agoraphobia – Adult (SMA-A; Craske *et al.*, 2013a); in the case of social anxiety, the Liebowitz Social Anxiety Scale (LSAS; Fresco *et al.*, 2001; Liebowitz, 1987); in the case of specific phobia, the Severity Measure for Specific Phobia - Adult (SMSP-A; Craske *et al.*, 2013b); in the case of generalised anxiety, the Generalised Anxiety Disorder-7 (GAD-7) ( Spitzer *et al.*, 2006); in the case of obsessive-compulsive disorders, the Yale-Brown Obsessive Compulsive Scale (Y-BOCS; Goodman *et al.*, 1989a; Goodman *et al.*, 1989b; Steketee *et al.*, 1996); and in the case of trauma/stressor related disorders, the PTSD Checklist for DSM-5 (PCL-5; Weathers *et al.*, 2013).

It is also envisaged that we will develop and use three experimental measures inspired by the Overall Other Emotion Severity and Impairment Scale, as used in the UP transdiagnostic treatment ( Barlow *et al.*, 2017b). These three experimental measures will be: the Overall Shame Severity and Impairment Scale (OSSIS), the Overall Loneliness Severity and Impairment Scale (OLSIS), and the Overall Fear Severity and Impairment Scale (OFSIS). These scales should tap onto participants’ underlying chronic painful feelings of shame, loneliness/sadness, and fear (see below). Initially, all three measures will be administered to all participants. Subsequently only measures on which a participant scored ≥8 (this tentative cut-off is based on the wording of the anchors and cut-offs for OASIS and ODSIS) will be used for that particular individual. At the end of active treatment, participants will be also interviewed using the Client Change Interview Protocol (CCIP; Elliott, 1999) which enquires about changes experienced by the individual since therapy started and helpful and unhelpful aspects of therapy.

**Figure 1.**
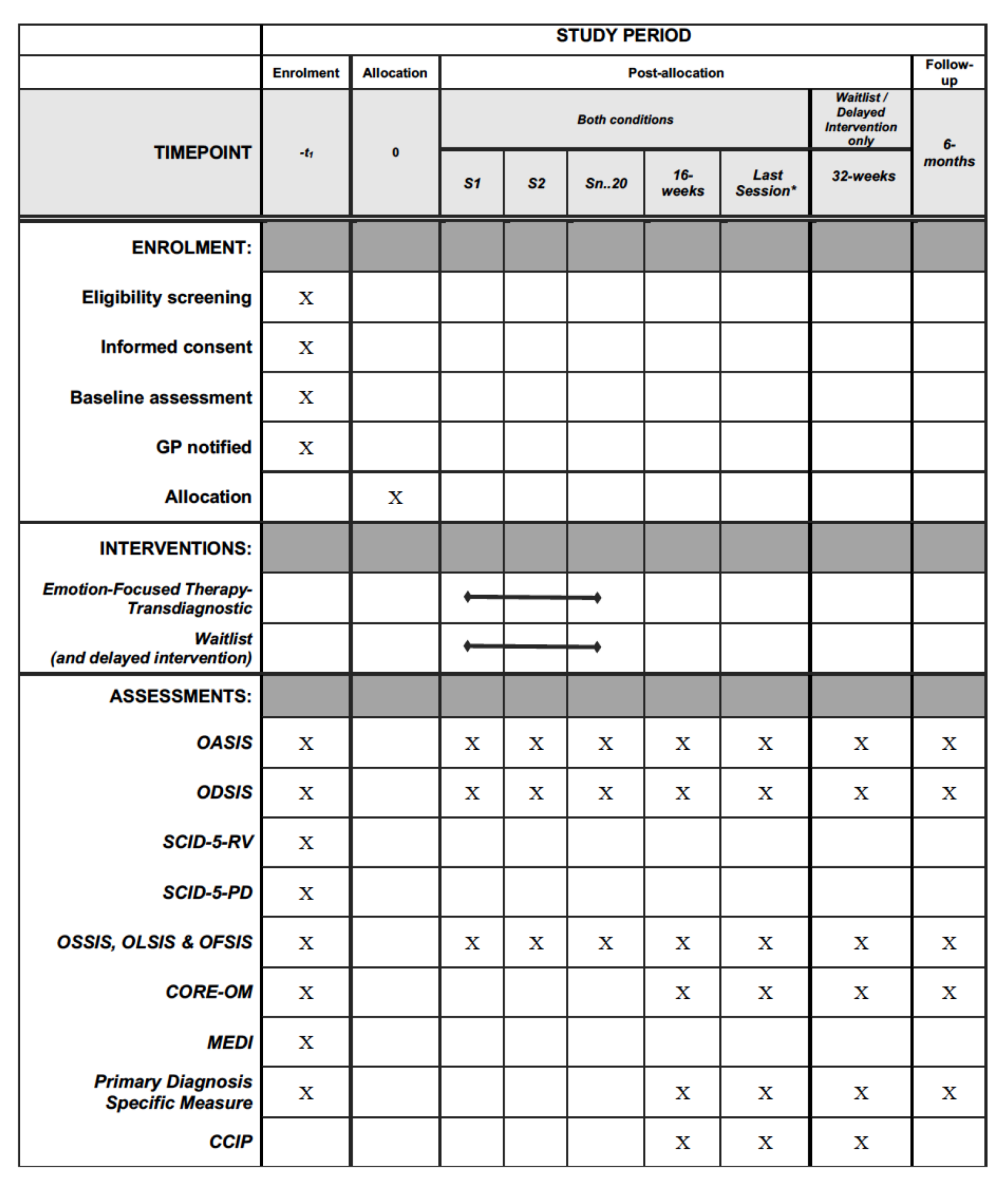
SPIRIT diagram of enrolment, intervention, and assessment. CCIP, Client Change Interview Protocol; CORE-OM, Clinical Outcomes in Routine Evaluation-Outcome Measure; MEDI, Multidimensional Emotional Disorder Inventory; OASIS, Overall Anxiety Severity and Impairment Scale; ODSIS, Overall Depression Severity and Impairment Scale; OFSIS, Overall Fear Severity and Impairment Scale; OLSIS, Overall Loneliness Severity and Impairment Scale; OSSIS, Overall Shame Severity and Impairment Scale; SCID-5-RV, Structured Clinical Interview for DSM-5 Disorders (Research Version); SCID-5-PD, Structured Clinical Interview for DSM-5 Personality Disorders. *Assessments are only administered at last session when therapy ends ≤ week 13 or ≥ week 19.

Those individuals who proceed to become study participants will be allocated a unique trial code, and all subsequent documents will be referenced by this code to protect participant confidentiality. It is anticipated that the first therapy session for participants in the active condition will typically take place one week after the second assessment appointment with the research team. Prior to each therapy session, participants will be asked to complete the OASIS, ODSIS and any of the three experimental measures (OSSIS, OLSIS, and OFSIS) on which they scored ≥8 at pre-therapy. Post-treatment assessments will take place at week 16 (i.e., as close as possible to 16 calendar weeks from the date of the first session) and 6 months post 16 weeks (as close as possible to 42 calendar weeks from the date of the first session). In addition, where participants finish therapy outside the range of 16 ± 2 weeks (i.e., ≤ 13 weeks, or ≥ 19 weeks), an additional assessment will be carried out post therapy (i.e., as close as possible to the date of the last session). The 16-week, post-therapy and 6-month follow up assessments will consist of administering the ODSIS, OASIS, and CORE-OM (these are administered to all participants), a diagnosis specific measure (selected for each participant on the basis of their primary diagnosis), and any of the three experimental measures (OSSIS, OLSIS, and OFSIS) on which a participant scored ≥8 at pre-therapy. The qualitative interview (CCIP) will be conducted only post-treatment. A summary of the assessments is presented in Figure 1.

**Figure 2.**
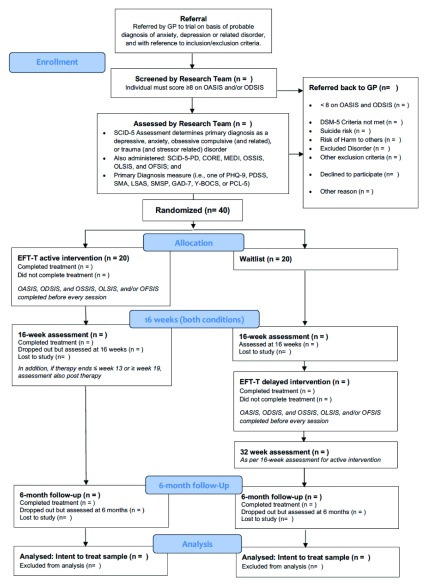
Study flow diagram of referral, screening and allocation of patients to the EFT-T study. Client Change Interview Protocol; CORE-OM, Clinical Outcomes in Routine Evaluation-Outcome Measure; DSM, Diagnostic and Statistical Manual of Mental Disorders; MEDI, Multidimensional Emotional Disorder Inventory; GAD-7, Generalized Anxiety Disorder-7; LSAS, Liebowitz Social Anxiety Scale; OASIS, Overall Anxiety Severity and Impairment Scale; ODSIS, Overall Depression Severity and Impairment Scale; OFSIS, Overall Fear Severity and Impairment Scale; OLSIS, Overall Loneliness Severity and Impairment Scale; OSSIS, Overall Shame Severity and Impairment Scale; PCL-5, PTSD Checklist for DSM-5; PDSS, Panic Disorder Severity Scale; PHQ-9, Patient Health Questionnaire-9; ; SCID-5-RV, Structured Clinical Interview for DSM-5 Disorders (Research Version); SCID-5-PD, Structured Clinical Interview for DSM-5 Personality Disorders; SMA, Severity Measure for Agoraphobia; SMSP, Severity Measure for Specific Phobia; YBOCS, Yale-Brown Obsessive-Compulsive Scale.

If, during the course of the treatment, any client’s clinical condition suggests another course of treatment (e.g., further assessment, hospitalisation, acute risk management) this will be provided as per typical clinical considerations. The clinical lead (JMcE) and/or the PI (LT) will oversee any clinical issues arising. All efforts will be made to carry out 16-week and 6-month follow up assessments with all participants irrespective of whether they complete or drop out of therapy. Figure 2 presents a flow study chart of the progress of participants though the study.

### Measures

***Assessment of participants.*** Potential participants will be assessed for principal diagnosis of depression, anxiety and/or related disorders as well as any other co-morbid condition using the SCID-5-RV and the SCID-5-PD. The MEDI, an emerging tool used in transdiagnostic research will also be administered. Demographic data as well as data related to presenting issues will be collected.

**SCID-5-RV.** The SCID-5 ( First *et al.*, 2015) is a semi-structured diagnostic interview for assessing the major DSM-5 diagnoses (formerly conceptualised as Axis I disorders). Presently, there is no reliability or validity data available for the SCID-5. However, many reliability studies of previous SCID versions have been published, typically reporting fair to excellent levels of agreement (e.g., Lobbestael *et al.*, 2011). Regarding validity, the SCID is often used as the gold standard in determining the accuracy of clinical diagnoses (e.g., Basco *et al.*, 2000).

**SCID-5-PD.** The SCID-5-PD ( First *et al.*, 2016) is a semi-structured diagnostic interview for assessing the 10 DSM-5 personality disorders in clusters A, B, and C. Presently, there is no reliability or validity data available for the SCID-5. Based on previous studies, First et al. (2016) observed that with both the SCID-5 and SCID-5-PD, reliability is likely to be higher in cases of joint interviewing, where raters are well trained, where presentations are more severe, and where diagnoses have higher base rates in the population.

**MEDI** The MEDI ( Rosellini, 2013; Rosellini *et al.*, 2015) is an experimental self-report questionnaire, still in development, which aims to assess transdiagnostic vulnerabilities and phenotypes that would allow a profile (as opposed to categorical) approach to emotional disorder classification. Participants are asked to score 55 items using a response scale that ranges from 0 (not characteristic of me/does not apply to me) to 8 (extremely characteristics of me/applies to me very much) resulting in composite scores on eight subscales; neurotic temperament, positive temperament, depressed mood, autonomic arousal, somatic anxiety, social evaluation concerns, traumatic re-experiencing and dissociation, and avoidance. A pilot study has reported convergent and discriminant validity of the eight MEDI subscales compared to other well-validated self-report questionnaires ( Rosellini, 2013).

**Demographic data** These will be collected as part of the pre-trial assessment and will include data regarding age, gender, relationship status, living arrangements, number of dependents, level of education attained, occupation and work history, and disability.

**Data related to presenting issues** As part of the same assessment process, a clinical interview will gather data about the history of the client’s presenting and other psychological difficulties, including past and current interventions, medication, suicidality, substance use and any other potential risk factors.

***Primary outcome measures.*** Severity of depression and anxiety symptoms will be measured at pre-therapy, post therapy (16 weeks; but also post last session in cases where the last session falls outside the range of 16 ± 2 weeks) and at 6-month follow up, using the OASIS, ODSIS and CORE-OM as primary outcome measures. In addition, the OASIS and ODSIS will be administered at the beginning of every session, as they are similarly used in an established cognitive-behavioural transdiagnostic treatment (UP; Barlow *et al.*, 2017b). With the exception of where a participant has explicitly stated a wish to withdraw from the study, participants who drop out of therapy will be contacted by the research team and invited to attend for 16 weeks and 6-month assessments.

**OASIS** The OASIS ( Norman *et al.*, 2006) is a 5-item, continuous, self-report measure of anxiety-related severity and impairment designed for use across anxiety disorders as well as in cases of subsyndromal anxiety. Items ask about anxiety and fear as experienced by the respondent over the past week, and are scored on a five-point Likert scale ranging between 0 (None; Little to none) and 4 (Extreme; All the time), which are then summed to provide one total score. The scale has demonstrated excellent 1-month test-retest reliability, convergent and divergent validity ( Norman *et al.*, 2006) and strong sensitivity to change ( Norman *et al.*, 2013).

**ODSIS** The ODSIS ( Bentley *et al.*, 2014) is a 5-item, continuous self-report measure designed for use across heterogeneous mood disorders and with subthreshold depressive symptoms. Items ask respondents about their experience of depression over the past week, are scored on a five-point Likert scale ranging between 0 (None; Little to none) and 4 (Extreme; All the time) with scores summed to provide one total score. Good reliability and validity for the ODSIS has been reported ( Bentley *et al.*, 2014).

**CORE-OM** The CORE-OM ( Evans *et al.*, 2000) is a 34-item questionnaire measuring psychological distress across four domains; subjective well-being, problems or symptoms, life functioning and risk. Respondents are asked to score items in relation to how they have been feeling over the past week on a five-point Likert scale ranging between 0 (not at all) and 4 (most or all of the time), yielding four domain scores as well as overall total and total (minus risk) scores. Good internal and test-retest reliability, and good convergent validity with other measures of psychological distress have been demonstrated ( Evans *et al.*, 2002).

***Secondary outcome measures.*** Each participant will also be asked to complete at pre-therapy, post therapy (16 weeks; but also post last session where the last session falls outside the range of 16 ± 2 weeks) and at 6-month follow up, one of the following disorder specific measures (PHQ, PDSS, SMA-A, LSAS, SMSP-A, GAD-7, YBOC, or PCL-5), as determined by the principal diagnosis assessed at pre-therapy. Additionally, three newly developed measures, the OSSIS, OLSIS, and OFSIS, will be administered during the pre-therapy assessment, with those measures on which a client scores 8 or higher, thereafter administered prior to each session, post-therapy and at follow-up.

**PHQ-9** The PHQ-9 ( Kroenke *et al.*, 2001) is a nine-item self-report instrument intended to assess the existence and severity of symptoms of depression. Internal reliability of the PHQ-9 has been reported as excellent, while construct validity, external validity and test-retest reliability have all been satisfactory. A clinical cut-off score of ≥10 has been suggested ( Kroenke *et al.*, 2001).

**PDSS** The PDSS ( Shear *et al.*, 1997) is a 7-item self-report scale comprised of five items assessing the core symptoms of DSM-IV defined panic disorder, with or without agoraphobia, and two additional items rating work and social impairment. Items are scored on a 5-point Likert scale. Excellent interrater reliability, moderate internal consistency, and favourable levels of validity and sensitivity to change have been reported. A cut-off score of 8 has been recommended as identifying the presence of current Panic Disorder ( Shear *et al.*, 1997; Shear *et al,*, 2001).

**SMA-A** The SMA-A ( Craske *et al.*, 2013a) is a 10-item measure that assesses the severity of symptoms of agoraphobia in individuals age 18 and older over the past 7 days. Items are scored on a 5-point Likert scale with average scores of 1 – 4, respectively indicating mild, moderate, severe, and extreme severity of agoraphobia. The use of the average total score was found to be reliable, easy to use, and clinically useful in American Psychiatric Association (APA) DSM-5 field trials.

**LSAS** The LSAS ( Liebowitz, 1987) is a 24-item scale designed to assess the range of social interaction and performance situations that individuals with social anxiety may fear or avoid. Good reliability and convergent validity have been reported for the self-report measure ( Fresco *et al.*, 2001). A cut-of score of 30 has been recommended as identifying the presence of SAD ( Mennin *et al.*, 2002).

**The Severity Measure for Specific Phobia – Adult (SMSP-A)** The SMSP-A ( Craske *et al.*, 2013a) is a 10-item measure that assesses the severity of symptoms of specific phobia in individuals age 18 and older over the past 7 days. The measure was designed to be completed by an individual upon receiving a diagnosis of specific phobia (or clinically significant specific phobia symptoms) and thereafter, prior to follow-up visits with clinicians. Items are scored on a 5-point Likert scale with averaged scores of 1 – 4 respectively indicating mild, moderate, severe, and extreme severity of specific phobia related distress. The use of the average total score was found to be reliable, easy to use, and clinically useful in APA DSM-5 field trials.

**GAD-7** The GAD-7 ( Spitzer *et al.*, 2006) is a 7-item self-report questionnaire assessing GAD symptoms over the preceding two weeks. Using the threshold score of 10, the GAD-7 has been reported to have a sensitivity of 89% and a specificity of 82% for GAD; it has also been reported as having good reliability, as well as criterion, construct, factorial, and procedural validity ( Spitzer *et al.*, 2006).

**The Yale-Brown Obsessive Compulsive Scale (Y-BOCS)** The Y-BOCS ( Goodman *et al*., 1989a; Goodman *et al*., 1989b) lists 58 separate obsessions and compulsions and asks subjects to indicate whether they have experienced any of these in the past or experience any of these currently. Subjects are asked to identify the two most currently experienced upsetting obsessions and the two most upsetting compulsions. They are then asked to rate on a 5-point Likert scale a further 11 items relating to their experience of these obsessions and compulsions over the past 7 days. The scale yields an overall score, as well as scores on separate Obsessions and Compulsions subscales. The Y-BOCS can be completed by a clinician as part of a clinical interview or can be completed as a self-report measure. As a self-report measure, the Y-BOCS has been reported as showing excellent internal consistency and test-retest reliability ( Steketee *et al.*, 1996).

**PCL-5** The PCL-5 ( Weathers *et al.*, 2013) is a 20-item self-report instrument designed to assess symptoms of posttraumatic stress disorder (PTSD). It is an updated version of the PTSD Checklist, revised to take into account changes to the diagnosis of PTSD in DSM-5, and replacing both the military and civilian versions of the earlier measure. Respondents indicate the degree to which they were bothered by posttraumatic symptoms in the previous month using a 5-point Likert-type scale ranging from 1 (not at all) to 5 (extremely). Strong internal consistency test-retest reliability, and convergent and discriminant validity has been reported for the PCL-5 ( Blevins *et al.*, 2015).

**OSSIS, OLSIS and OFSIS** The OSSIS, OLSIS, and OFSIS are three new experimental self-report measures which will be developed as part of this study. Emotion-Focused Therapy proposes that clients' symptomatic presentations are underpinned by maladaptive shame-based, loneliness-based and fear-based emotion schemes, and that changes in these schemes over time should correlate with changes in symptomatic distress. Modelled on the Overall Other Emotion Severity and Impairment Scale developed by Norman *et al.* (2006), these scales will be 5-item, continuous, self-report measures of maladaptive shame, loneliness and fear as experienced by the respondent over the past week.

***Qualitative measure***

**CCIP** The CCIP ( Elliott, 1999) is a structured interview that asks client about their experience of change (including change for the worse) since the beginning of therapy. Clients are asked for their perspective on those changes. They are also asked about helpful and problematic aspects of therapy.

### Assessors

Assessments will be conducted by doctoral level psychologists and by psychology graduates (typically at master’s level) under the supervision of a doctoral level psychologist. The SCID-5-RV and SCID-5-PD assessments will be audio recorded and archived for subsequent evaluation of adherence to appropriate testing conditions. All assessments including post-therapy self-report assessments will be conducted by a clinician other than the therapist.

### Sample size

A sample size of 40 participants was determined (using G*Power – Faul *et al.*, 2007) on the basis of the comparison between and within the two groups across time, and allowing for research attrition. Moderate effect size (f= 0.25) was used as a determinant of meaningful between groups difference with statistical power of 0.80 and alpha level of 0.05. It is expected that the study is likely to detect change over time within the active treatment (EFT-T) and in-between the active treatment and the wait-list. As the current project is a feasibility study, exploratory use of these initial data should help in planning a definite comparative non-inferiority trial, comparing EFT-T and a standard transdiagnostic CBT.

### Data management

On referral to the project all potential participants will be given a referral code. Each participant who proceeds to pre-trial assessment by the research team will be given a unique study code which identifies the client as assessed for the study. Participants who proceed from assessment to the trial will then be given a unique trial code, which will identify the client and the corresponding therapist. Paper copies of all measures will be identifiable only by the study and/or trial codes.

All data will be stored in locked filing cabinets in locked offices in the counselling centre and/or the School of Psychology, Trinity College Dublin. All identifying paper data (e.g., GP referral information, signed consent forms; and forms listing participant codes) will be stored in a separate locked filing cabinet to all other anonymised data, and will be accessible only to the Trial Manager and to members of the research team involved in pre-therapy and post-therapy assessments. All audio/video and electronic data will be stored on encrypted hard drives.

In order to mitigate against data entry errors, all data will be double entered into two parallel datasets by two separate members of the research team, and the two datasets will be routinely audited and compared. Multiple imputation will be used for missing data. All regulations set by the Research Ethics Committee at the School of Psychology, TCD, as well as data protection regulations will be observed.

### Statistical methods

The main analysis will be run as intent-to-treat analysis (IIT) (within and between groups comparisons at 16 weeks, and the end of treatment) as well as per-protocol analysis (PP; which will exclude participants who had less than 8 sessions – i.e., half of the expected length of the treatment). Primary (OASIS, ODSIS, and CORE-OM) and secondary outcomes (client relevant disorder specific measures; as well as OSSIS, OLSIS, and OFSIS) will be analysed using repeated measures ANOVAs at 16 weeks and at the end of treatment. Effects will be tested at the 0.05 level. After 16 weeks, participants from the wait-list will be provided with the active treatment, and pre-post and 6 months follow-up will be assessed for the collated group. Analysis will also be conducted to determine the proportion of participants who achieved clinically significant change and reliable improvement ( Jacobson & Truax, 1991) at the end of the treatment, at 16 weeks, and at 6 months follow-up. The size of the within-group effects (Cohen’s *d*) will be calculated. The magnitude of between-group effects at 16 weeks and at the end of treatment will also be established.

### Governance and oversight of the trial

The trial management group (TMG) will be concerned with the day-to-day operations of running the trial and will monitor all aspects of the project. The TMG will meet approximately every two to three months. Membership will consist of the principal investigator (PI) and EFT trainer and supervisor (LT), the trial manager (DK), and the clinical lead (JMcE). The group will regularly discuss issues such as recruitment of participants, retention of participants, clinical issues that are arising, clinical governance and ethical issues, adverse events or other unintended effects of trial interventions or trial conduct, procedures for pre-therapy, post-therapy and follow up assessments, data management processes, data analysis, and any other issues that arise. Any potential changes to the trial protocol will be discussed, including any potential reasons to discontinue the trial, and procedures for disseminating decisions to relevant parties will be specified, including bringing any changes to the trial steering committee (TSC).

The TSC will meet every 6 months over the course of the project and will have the function of independently overseeing the project including the review of ethical issues, changes to protocol, adverse events or other unintended effects of trial interventions or trial conduct. Membership will consist of an independent chairperson, the above-mentioned members of the TMG, and optimally, a representative from the public. The PI will report to the committee on progress regarding the trial and seek lay/expert perspectives and queries from the group regarding any issues arising.

### Ethics approval

Ethical approval for this study was granted by the School of Psychology Research Ethics Committee, Trinity College Dublin, Dublin, Ireland on 14/12/2018.

## Discussion

Depression, anxiety and related disorders such as obsessive-compulsive disorders and trauma/stressor related disorders ( APA, 2013) represent the majority of presentations typically seen in an outpatient setting that are treated by psychological therapies. While depression, anxiety and related disorders represent distinct diagnostic categories, they have very high comorbidity. Furthermore, they share genetic vulnerability, neurobiological mechanisms, developmental risk factors and underlying psychological mechanisms. In addition, psychological treatments for a single diagnostic category positively impact on present co-morbid conditions.

The above factors have led to the development of transdiagnostic, predominantly CBT, psychological treatments. Developing non-CBT transdiagnostic treatments should contribute to patients’ choice in treatment. EFT was developed as a treatment using universally applicable principles to various client presentations. Furthermore, it was specifically adapted for depression, generalized anxiety, social anxiety, and complex trauma. This study aims to provide an initial test of EFT-T as a transdiagnostic treatment for depression, anxiety and related disorders. It will provide estimates of effects sizes that can inform power calculations for a comparative trial, comparing EFT to a standard transdiagnostic treatment, CBT. As a feasibility study, it should contribute to the planning of a definitive, non-inferiority trial that would establish EFT’s relative efficacy in comparison to a transdiagnostic CBT as an established treatment. Depending on the results of this feasibility study, we should also be able to assess the practical issues involved in running such a design. The qualitative part of the study will give voice to participants and will allow us to know more about the clients’ experiences of therapy (both helpful and unhelpful) and of any changes that may have happened since beginning therapy. Clients' perspectives can thus further inform future adaptations of the treatment.

### Future availability of data and materials

Once results have been published in peer-reviewed academic journals, relevant data and materials can be made available on request to other researchers via the Trinity College Dublin TARA data repository, in keeping with Trinity College Dublin research policy.

### Trial status

Recruitment of participants commenced in February 2019. The approximate trial duration is 36 months. The trial was registered with the ISRCTN registry on January 2nd 2019 ( http://www.isrctn.com/ISRCTN11430110).

## Data availability

### Underlying data

No underlying data are associated with this article.

### Extended data

Harvard Dataverse: EFT-T vs Waitlist feasibility RCT Documents. https://doi.org/10.7910/DVN/Z8ZESJ ( Timulak, 2020).

This project contains the following extended data:

- Debriefing Form (EFT-T Study). (Debriefing form given to participants.)
- GP Information Leaflet (EFT-T Study).
- Participant Confidential Info (EFT-T Study). (Form containing confidential participant data e.g., name and contact details).
- Participant Consent Form (EFT-T Study).
- Participant Data Summary Form (EFT-T Study).
- Participant Information Leaflet (EFT-T Study).

### Reporting guidelines

Harvard Dataverse: SPIRIT checklist for ‘Emotion-focused therapy as a transdiagnostic treatment for depression, anxiety and related disorders: Protocol for an initial feasibility randomised control trial’. https://doi.org/10.7910/DVN/Z8ZESJ ( Timulak, 2020).

Data are available under the terms of the Creative Commons Zero "No rights reserved" data waiver (CC0 1.0 Public domain dedication).

## Author contributions

LT conceived and designed the study, and is principal investigator. He is responsible for the overall running of the project and for drafting the trial protocol. He is also responsible for selecting EFT certified therapists and for training them in the protocol. DK is the trial manager, was responsible for the ethics submissions, co-drafted the trial protocol and created the SPIRIT checklist. JMcE is responsible for issues of clinical governance pertaining to the trial. SS, CJ, and KT will contribute to participant recruitment and day-to-day running of the trial. NH will share responsibility for the training and supervision of therapists. FW contributes to planning and oversight of the trial via participation in the Trial Management Group and Trial Steering Committee.
