# Supplementary material for: Emotion-focused therapy as a transdiagnostic treatment for depression, anxiety and related disorders: Protocol for an initial feasibility randomised control trial
Source: HRB Open Res. 2020 Feb 13;3:7. [Version 1] doi: 10.12688/hrbopenres.12993.1 (PMC7140776; doi:10.12688/hrbopenres.12993.1)
Supplement: Peer review history

# Peer review: Reviewer response for version 1

**DOI:** 10.21956/hrbopenres.14083.r27183

**Published:** 2020-03-10

**Authors:** Carla Cunha

**Affiliations:** 1 Center for Psychology at University of Porto, Porto, Portugal; 2 Department of Social and Behavioural Sciences, University Institute of Maia - ISMAI, Maia, Portugal

This Study Protocol paper is very well written, providing a strong rationale for the pertinence of developing and testing an Emotion-focused therapy transdiagnostic treatment for depression, anxiety and related disorders. The authors provide a clear explanation for focusing on developing new transdiagnostic treatments, adequately recognizing the need to address the pressure that is put on health professionals for achieving high proficiency in a plethora of single-disorder treatments, while most of the clinical reality of their clients is related to co-morbid disorders. Their overall purpose to depart from this feasibility study to set the stage for a future randomised clinical trial that would establish the relative efficacy of Emotion-focused therapy transdiagnostic treatment compared to a transdiagnostic CBT treatment is also highly recommendable and an example of golden standard research practice (Eldridge, Lancaster, *et al*., 2016 ^1^).

 The Abstract and Introduction present a compelling argument for the advantages of transdiagnostic treatments and, in my view, this study presents a valuable contribution to this field, namely by developing an alternative to transdiagnostic treatments that essentially come from Cognitive-Behavioral Therapy, which “is not the preferred treatment to all clients, nor do all clients benefit from”, as the authors highlight on page 3. On this regard, I encourage authors also to emphasize prior research concerning the positive impact of attending to client treatment preferences, given that it affects treatment satisfaction, completion, and clinical outcome (see the meta-analysis by Lindhiem, Bennett, Trentacosta, & McLear, 2014 ^2^).

 After reading this Study Protocol, it is my view that it adequately addresses what is expected in terms of this type of article (Study Protocol), complying with the CONSORT statement for feasibility trials (Eldridge, Chan, *et al*., 2016 ^3^), both in terms of content and figures (1 and 2). The study description, method, design and measures are well justified and ensure replicability for future, similar studies. Furthermore, the procedures related to treatment fidelity, randomisation and data analysis are in line with this study goals as a feasibility trial (treatment adherence, retention rates, etc.). The procedures related to gathering client consent, data management and governance/oversight of this trial and securing ethics approval, seem adjusted to current standards for research.

 I only have a few minor comments/suggestions to address:

- The acronym EFT-T is usually known in the literature as Emotion-Focused Therapy for Trauma (EFTT), referencing the work by Sandra Paivio and colleagues (e.g. Paivio & Angus, 2017 ^4^; Paivio & Pascual-Leone, 2010 ^5^). To avoid the confusion of using two very similar acronyms, I would suggest authors to choose a slightly different way to refer to the Emotion-focused therapy transdiagnostic treatment, perhaps through “t-EFT”, “T-EFT”  or another alternative.
- On page 3, the reference Brown *et al*. (2001) for current and lifetime prevalence of co-morbidity between seems accurate but outdated.

I confirm that I have read this submission and believe that I have an appropriate level of expertise to confirm that it is of an acceptable scientific standard.

## References

[1] : Defining Feasibility and Pilot Studies in Preparation for Randomised Controlled Trials: Development of a Conceptual Framework. *PLoS One*.2016;11(3) : 10.1371/journal.pone.0150205 e0150205 10.1371/journal.pone.0150205 26978655PMC4792418

[2] : Client preferences affect treatment satisfaction, completion, and clinical outcome: a meta-analysis. *Clin Psychol Rev*.2014;34(6) : 10.1016/j.cpr.2014.06.002 506-17 10.1016/j.cpr.2014.06.002 25189522PMC4176894

[3] : CONSORT 2010 statement: extension to randomised pilot and feasibility trials. *BMJ*.2016;355: 10.1136/bmj.i5239 i5239 10.1136/bmj.i5239 27777223PMC5076380

[4] : Narrative processes in emotion-focused therapy for trauma. 2017; 10.1037/0000041-000 10.1037/0000041-000

[5] : Emotion-focused therapy for complex trauma: An integrative approach. 2010; 10.1037/12077-000 10.1037/12077-000

# Peer review: Reviewer response for version 1

**DOI:** 10.21956/hrbopenres.14083.r27179

**Published:** 2020-03-04

**Authors:** Eva Dubovská

**Affiliations:** 1 Olomouc University Social Health Institute, Palacký University Olomouc, Olomouc, Czech Republic

The present paper presents a plausible protocol for the randomized controlled trial to establish the efficacy of EFT-T as compared to wait list control condition, for mixed diagnoses of depression, anxiety, trauma and OCD. It may provide valuable data to enable comparison with the dominant transdiagnostic approach, which is CBT. This will be an important study that enables further research on EFT and transdiagnostic treatments. Given the high prevalence of comorbidities within these patient groups, the transdiagnostic approach may be a promising way to address psychological processes, that are common to above mentioned disorders.

 All steps of the study are described in a comprehensive way. At the entry level an SCID-5 assessment will be used to determine the primary diagnoses and as main outcome measures the OASIS, ODSIS and CORE-OM self-reports, but also complementary primary diagnoses measures such as PHQ-9, PDSS, LSAS, SMA etc. will be administered. A 6-month follow-up is planned after the end of treatment.

 Another strength of this protocol is that three new EFT specific experimental measures will be specifically developed as part of this study (OSSIS, OLSIS, OFSIS). There is also space for subsequent qualitative research based on the Client Change Interview protocol. This design has potential, combined with the analysis of the session recordings, to bring some new original in-depth knowledge on the change process during the EFT treatment.

 Although the study protocol is very precise, I would welcome some more additional information regarding the planned data analysis, mainly what strategy will be used for the missing outcome data and possible exclusions from analysis with regard to proclaimed intent-to-treat analysis.

I confirm that I have read this submission and believe that I have an appropriate level of expertise to confirm that it is of an acceptable scientific standard.

# Peer review: Reviewer response for version 1

**DOI:** 10.21956/hrbopenres.14083.r27182

**Published:** 2020-02-26

**Authors:** Ueli Kramer

**Affiliations:** 1 Institute of Psychotherapy, Department of Psychiatry, Lausanne University Hospital, University of Lausanne, Lausanne, Switzerland

This is a convincing protocol for a feasibility trial involving the comparison of a transdiagnostic treatment - emotion-focused therapy - with wait-list control. Primary outcomes will be depression, anxiety (measured by OASIS, ODSIS and CORE-OM). Additional outcomes involve severity of depression, symptoms of panic disorder and agoraphobia and other phobias, generalized anxiety disorder, obsessive compulsive disorder and post-traumatic stress symptoms.

 The researchers outline a detailed assessment schedule which is both complete and bears all central aspects which pertain to the quantitative assessment of change in transdiagnostic treatment.

 In addition, treatment integrity, and client perspectives, and contributions are assessed using observer-rated methods and qualitative methods of investigation. As such, this study may lay the groundwork for a programme of research focusing on transdiagnostic treatments focusing on emotion.

 One challenge in this context is certainly the notion of “transdiagnostic” itself, and the fact that there may be several clinical problems which may require quite different treatment stances, or approaches. This is not to question entirely the approach, but rather to raise awareness that the treatment of symptoms of post-traumatic stress disorder may require quite different interventions than the treatment of an unipolar depression. While it is laudable to address the underlying emotional processes in each of these treatments (and they may look indeed similar), there will be a great between-subject variability which need to be attended to clinically, and statistically in the analyses. Relatedly, it remains unclear whether problems related with personality disorders, often found co-morbid with the disorders outlined in the protocol, will be included or not.

 All in all, a convincing and challenging protocol, one researchers and clinicians from various theoretical orientations will hopefully find interesting for their own work.

I confirm that I have read this submission and believe that I have an appropriate level of expertise to confirm that it is of an acceptable scientific standard.
